# Our Christmas Supplement

**Published:** 1903-12-12

**Authors:** 


### OUR CHRISTMAS SUPPLEMENT.

### OUR HOSPITALS.

### THE PRESENT POSITION AND OUTLOOK.

THE year now drawing to a close has been an eventful one in the history of the hospitals of London. It has witnessed the ripening to maturity of several great designs which have for some time past been held in contemplation; designs which, there is every reason to hope, may, when completed, render our system, whereby the sick poor are relieved in their hour of need by the active and voluntary service of their more fortunate fellows, the most perfect and beautiful organisation for the relief of the sick and suffering that the world has ever seen. Great and lasting institutions are not completed in a day, and it is only by noble self-sacrifice and persistent endeavour on the part of the present generation that our voluntary hospital system can be established on foundations which will stand firm so long as the nation shall endure. We have now reached the very climax in the struggle for maintaining that system, and it is the duty and privilege of this generation to render a successful issue absolutely certain, and to see to it that apathy and selfishness shall never in the history of the English people be mentioned among the characteristics of the present enlightened age. It is therefore essential for every single individual, be he never so poor or humble, to put his energies to the work and give what help in time or money he can afford to our hospitals to maintain them in the van of modern scientific progress. The public has in the past year witnessed appeals for support from a considerable number of institutions, including some of the largest and most important as well as many of the smaller ones, for large sums of money which are necessary for reconstructing the buildings according to up-to-date hygienic requirements, and for the expansion rendered essential by the rapid increase of the numbers of people requiring assistance.

Again, a remarkable feature of the past forty years has been the redistribution of the population of London. Owing no doubt to the rise of land values in the more central parts, together with improvements in the means of intercommunication between the different sections of the metropolis, the number of residents in the central districts has actually decreased, while the suburban population has increased with enormous rapidity. This circumstance has given evidence of the necessity for a reallotment of the hospitals in order that the poor may have the opportunity of receiving medical treatment and advice within a reasonable distance of their own homes. Certain of the hospitals have resolved to move further afield, prominent among which is King's College Hospital, the authorities of which have already been presented with a site of 12 acres in Camberwell, and are now asking for £300,000 to defray the cost of the move. As the removal of this institution will go a great way towards solving one of the most important and pressing hospital problems of the day by providing a large section of the population of South London with sorely needed facilities for medical treatment, and as the area of the site is sufficient to allow of the erection of a perfect modern hospital, there is every reason to believe that the appeal will meet with a hearty and generous response.

Early in the year the authorities of the London Hospital were pleading for a quarter of a million pounds, and many of the other hospitals were asking for special help, while in the ensuing year there is a prospect of even larger appeals, for the authorities of St. George's Hospital are at this moment considering the ways and means of finding a new home on a more extensive site than that which the hospital at present possesses, while St. Bartholomew's Hospital is about to make a big demand for assistance for purposes of reconstruction.

Those whose enthusiasm is only half-hearted will probably be somewhat appalled by the magnitude of the sums which the public are expected to supply out of their generosity. It will take probably ten years to raise the funds, but in the end they will no doubt be forthcoming. To all true lovers of charity these appeals will be welcome signs, for they not only bring the promise of greater efficiency and scope of usefulness, but they also show incidentally the faith which is placed in the benevolence of the present generation. Moreover, some useful lessons may be learned from these large demands. Had subscriptions been forthcoming in past years sufficient in amount to defray the ordinary expenditure of the hospitals all the moneys received from time to time in the form of legacies would have been available to meet the extraordinary expenditure as occasion arose. But this, unfortunately, has not been the case hitherto, and only too frequently bequests have been absorbed in payments which it were best, if it were possible, to meet out of the annual income. The consequence has been that when the time has arrived for necessary rebuilding and improvements there has been no capital in reserve to meet the additional outlay involved. If in the future everyone performs his share of the work a comparatively small effort will be sufficient to raise the annual income from subscriptions to the amount required, and all amounts received from legacies can be placed to a reserve fund to meet extraordinary expenditure as occasion arises. Fortunately, as time goes on it becomes more and more evident that the general public are recognising this fact and intend to fulfil with no niggard hand the charitable obligations which are placed upon them. Owing to the activity of the central hospital funds no one can now complain that he would be willing to assist but that opportunities are not brought in his way. The Hospital Sunday Fund and Hospital Saturday Fund, together with the King's Fund and its useful ally the League of Mercy, cannot fail, one or all of them, to appeal directly to every single home in London, from the highest to the lowest. The steady growth of these funds is indeed one of the most hopeful auguries for the future of our hospitals. It has often been said with regard to institutions that they must either grow or decay. There can be no standing still. No one is likely, in face of the above facts, to deny that the voluntary hospital system of London is in the most flourishing and promising period of expansion.

In conclusion, let each one remember that in giving help to the hospitals he will be taking a part in the building of a monument the most noble that any nation can produce—a memorial of all that is best in human nature and a structure which is the most glorious feature of the present era. The strain during the next few years will no doubt be severe, but by then all our London Hospitals should have been modernised and placed upon a new basis of efficiency sufficient to meet the requirements of treatment for some fifty years to come. During these fifty years the revenue of each great hospital should grow to the required extent to meet all outgoings each year and to leave a small margin to spare. Courage and continuous effort have therefore every prospect of making our voluntary hospitals permanently secure within a very few years.

### What Each Hospital Needs at the Moment.

In pursuance of our plan of taking the bouquet of charity as represented by the London hospitals, and dealing with each flower or hospital in order, we shall endeavour to bring out clearly what each hospital is doing, what its present financial position is, and what are its most pressing requirements, which a benevolent public can advantageously further by a contribution in cash. We hope that the large number of wealthy people who have made it a practice to preserve the Christmas Appeal Number of The Hospital for reference throughout the year may be considerably increased this Christmas, and that all who use this issue as a handy book of reference for the purposes of their charitable gifts will find the plan pursued this year so helpful as to induce them to contribute even more liberally than they have heretofore done to the relief of sickness and accident in the metropolis of the Empire.

The Distribution Committee of the Hospital Sunday Fund has always had behind it the full confidence of the public, and we have therefore decided to quote the figures from the particulars issued under its authority. It is commonly recognised that the great general hospitals are the backbone of our hospital system, and the following striking figures give (1) the number of beds, (2) the average number of beds daily occupied, and (3) the number of in-patients under treatment during 1902 in the greater hospitals of London—that is, those containing 150 beds and upwards.

| Name of Hospital. | No. of Beds. | Average No. of Beds daily Occupied. | No. of In-Patients Treated. |
|---|---|---|---|
| London Hospital ... ... | 790 | 682 | 12,529 |
| *St. Bartholomew's Hospital ... | 674 | 481 | 6,587 |
| Guy's Hospital ... ... ... | 652 | 485 | 7,606 |
| St. Thomas's Hospital ... ... | 586 | 455 | 6,991 |
| Middlesex Hospital ... ... | 296 | 260 | 3.847 |
| St. George's Hospital ... ... | 351 | 251 | 3,751 |
| Hospital for Consumption, Brompton ... ... ... | 315 | 257 | 1,282 |
| St. Mary's Hospital ... ... | 281 | 248 | 3,927 |
| Dreadnought (Seamen's) Hospital ... ... ... ... | 269 | 234 | 2,545 |
| Hospital for Sick Children ... | 252 | 181 | 2,236 |
| King's College Hospital ... | 223 | 180 | 2,613 |
| Westminster Hospital ... ... | 213 | 158 | 2,384 |
| National Hospital for Paralysed | 196 | 171 | 1,004 |
| University College Hospital ... | 188 | 168 | 2.693 |
| Royal Free Hospital ... ... | 170 | 144 | 2,157 |
| City of London Hospital for Diseases of the Chest ... | 164 | 124 | 927 |
| Great Northern Central Hospital | 159 | 137 | 1,941 |
| Royal National Hospital for Consumption ... ... ... | 154 | 143 | 760 |
| Charing Cross Hospital... ... | 150 | 132 | 1,986 |

* Latest figures known 1901.

The figures which are given above indicate that the London Hospital passes many more in-patients through its beds than any other great hospital. What is the explanation of this? Are the patients discharged to a convalescent institution, or how is it managed?

### Hospitals with 150 Beds and Upwards.

### THE LONDON HOSPITAL.

This hospital is situated in the East End, where its influence for good is not confined to the medical relief it affords, for the humanising and civilising influences which result to a patient from a residence within its walls are of immense value to the citizens by raising the character of large numbers of the population throughout one of the poorest districts of London. This may also be said with truth in regard to other hospitals, but the influence in the case of the London is patent and appreciable owing to the vast numbers handled every year. During the last three years the average gross annual income has been

£98,398, and the average gross annual expenditure £112,231, showing a deficiency of about £14,000. Mr. Roberts has left, and is now secretary of St. Thomas's Hospital. It would be a calamity to East London if the work of this noble institution had to be curtailed. Its present circumstances justify special help, and even the £130,000 given by a generous public in answer to the Quinquennial Appeal of 1903 will fall very far short of meeting its immediate and pressing requirements and furnishing a sufficiency for its future upkeep.

### ST. BARTHOLOMEW'S HOSPITAL.

THIS is the only hospital in the country which is able to meet the whole of its expennditure out of the revenues derived from its invested property and assets. It has acquired a little more than an acre of ground from the trustees of Christ's Hospital, at a cost of about £250,000. Although within living memory it has never issued an appeal to the public for funds, St. Bartholomew's will no doubt appeal for a Building and Purchase of Further Land Fund to the extent of £600,000. If it does it ought and there is small doubt will receive ultimately this large sum, for it is the only general hospital in the City of London.

### GUY'S HOSPITAL.

AT the annual general Court of Governors held on November 6th, 1903, the serious financial condition of this institution came under consideration. It was stated that all the wards are now opened, more than 600 beds being available for patients, and that the cost of maintenance will not be less than £60,000 per annum. Towards this the hospital possesses an income, more or less assured, of some £35,000 per annum, and, in addition, has recently been in receipt of about £11,000 annually from King Edward's Hospital Fund for London, the Hospital Saturday and Sunday Funds, and subscribers, leaving a deficiency in ordinary income of £14,000 per annum. Of the £180,000 appealed for in 1901, on renovation and building account, only £103,000 has been received to date, and for the balance the institution is in debt on temporary loans. The Court unanimously resolved that:—"The treasurer be requested to obtain the widest currency possible for a statement of the present circumstances of the charity and further to continue a vigorous appeal, with a view of enlarging the basis of public support adequately to the increased needs of the establishment." Donations or annual subscriptions will be gratefully received by the Treasurer, Guy's Hospital, London Bridge, S.E.

### ST. THOMAS'S HOSPITAL.

THIS is one of the best known of our hospitals, owing to its situation on the Albert Embankment, where it occupies one of the most splendid sites in London. Six thousand nine hundred and ninety-one new cases and 67,409 out-patients were admitted for treatment during last year. The gross annual income, including legacies, amounted to £67,943, and the gross expenditure to £98,472. The deficiency of £30,500 was met by the sale of stock. About £12,000 per annum, from subscriptions, donations, and legacies, is essential for the upkeep of the hospital. Contributions will be gratefully received by the treasurer, Mr. J. G. Wainwright, who for a large number of years has rendered yeoman service to the institution. The present works in hand at St. Thomas's Hospital are: (1) certain reconstruction works connected with the in- and out-patients' departments, (2) a new nurses' home, and (3) a new power station. Contributions to the reconstruction fund will be gladly accepted, especially as some misapprehension appears to have arisen in regard to a recent legacy which will not, we understand, be paid for some time to come.

### THE MIDDLESEX HOSPITAL.

THE Middlesex Hospital, which is under the patronage of his Majesty the King and of her Majesty Queen Alexandra, is amongst the leading hospitals of the metropolis; but though one of the earliest general hospitals in London to be maintained by voluntary contributions, it has now only a very small endowment. There are 345 beds, all free, and during 1902 there were 4,019 in-patients and 41,585 out-patients. The recent extension of the nurses' home affords accommodation for a considerable addition to the nursing staff, a want which, although involving increased expenditure, was necessitated by the additional number of in-patients. This extension has cost £10,000. A special operating theatre for gynæcological cases has been constructed and equipped at a cost of £1,600. The basement, which has been set free by the removal of the laundry to Hendon, and of the kitchen to the first floor of the hospital, has been entirely remodelled, and is being fitted up with cold storage, disinfecting apparatus, and other domestic arrangements at a cost of £2,000. The cancer wards afford accommodation for 36 female and nine male patients, and during 1902 172 patients were received. The cancer research laboratories have been enlarged and an additional pathologist added to the staff, so that the work carried on there can be still more efficiently and satisfactorily performed. The convalescent home at Clacton-on-Sea, containing 70 beds, continues to prove invaluable for the rapid recovery of patients requiring good food and rest after discharge from the wards, and the "open-air" pavilion for the treatment of tubercular disease gives every indication of complete success. The ordinary income requires augmenting by £12,000 and £50,000 are needed to effect necessary improvements and to meet the liabilities which have been incurred. Secretary-superintendent, Mr. F. Clare Melhado.

### ST. GEORGE'S HOSPITAL.

THE finances of this institution, considered in conjunction with the fact that it is situated in the immediate proximity to the wealthiest quarter of London, cannot fail to provide material for grave meditation. During the last three years the average gross annual income has been £34,195, and the average gross annual expenditure £45,602, showing a deficiency of upwards of £12,000. Its pressing requirements are:—(1) a considerable expenditure upon the buildings and equipment of the hospital, and (2) the awakening of the wealthy residents around the hospital to the fact that its income is too small for the work it annually does, to so large an extent in their especial interest. Last year a special appeal was made, but was very poorly responded to, less than £8,000 being received as the result. Formerly St. George's Hospital had the finest revenue from annual subscriptions of any London hospital, but year by year there has been a gradual diminution in this important source of income, which would be unaccountable, and which we make bold to say would not even exist, if the subscriptions had been given by the relatively poorer residents to a hospital in a crowded district of London. St. George's Hospital occupies the most conspicuous site of any London hospital—a fact which, when taken in connection with the gradual falling off in the revenue from annual subscriptions, must be accepted as an indication that the popular belief that it is desirable, for financial reasons, that the site of a big hospital shall be in a leading thoroughfare, is largely erroneous. The question of removing the hospital to a new and larger site has been occupying the attention of the Governors during the past year, and a committee is at the present time engaged in considering the important matter of sites. Mr. Timothy Holmes is the Treasurer and Mr. C. L. Todd the Secretary.

### THE HOSPITAL FOR CONSUMPTION, BROMPTON.

PHTHISIS is the scourge of the human race, and the consumption hospitals, and especially that at Brompton, should receive the liberal support of all thoughtful people. It is probable that no institution has a greater or more pressing demand upon its beds than the Brompton Hospital. It administered to 1,545 in-patients and 12,924 out-patients last year. For the last three years the average gross annual income was £31,734, and the average gross annual expenditure £34,281, showing a deficiency of about £2,500. A most important addition to the relief afforded by this hospital is being provided in a country home, suitably situated on the Chobham Ridges, Surrey, where the open-air treatment will be available for suitable cases. The incalculable benefit this will confer on those of the poor who are afflicted with consumption should secure for it the ready and liberal support of the benevolent. The cost of this new departure will be £60,000, most of which has still to be contributed. Secretary, Mr W. H. Theobald.

### ST. MARY'S HOSPITAL.

PADDINGTON, W.

THIS hospital ministers to the needs of the poor scattered over the large area included in Paddington and the neighbouring districts. Last year it relieved 45,171 out- and midwifery patients in addition to 3,927 in-patients. It has always enjoyed a wide and deserved popularity, but does not receive an adequate measure of support from those who reside within the area which it serves. When it is stated that Paddington, Kensington, and Marylebone are the parishes whence the great majority of St. Mary's Hospital patients come it cannot be urged that the small proportion of well-to-do residents in those parishes is the reason for the small return which is made to the hospital for its good work among their poor neighbours in the hour of sickness, for these parishes are among the richest in London. They contain thousands of opulent residents to whom a three-guinea annual subscription to the hospital would be a mere trifle. Yet the total of annual subscriptions received by St. Mary's Hospital in the year is only £5,000, while its necessary annual expenditure is £30,000. The consequence is it has to "kill the goose that lays the golden eggs," and spend all its legacies instead of investing them to create an income. Its financial position is, therefore, most precarious, and prompt and liberal help is necessary if this great hospital is to be maintained in efficiency. Secretary, Mr. Thomas Ryan.

### SEAMEN'S HOSPITAL SOCIETY.

THE work of the Seamen's Hospital Society has been carried on with much vigour during the past twelve months, over 23,000 sick and injured seamen having been treated. The new out-patient department at the branch hospital in the Royal Albert Docks is approaching completion and will probably be occupied by the beginning of the year. It is with a sense of relief that the Committee will close the old wooden shed in which the work of treating out-patients has been carried on for so long. This necessary improvement will cost about £5,000, towards which very little has been received up to the present. Everbody in Great Britain owes a debt of gratitude to the sailor for most of the necessaries of life and nearly all the luxuries are brought to this country in ships, while there is no section of the community which stands more in need of succour and assistance when ill. As a rule the sailor is a stranger in the port without any relatives or friends to aid him, and it is to the various establishments of the Seamen's Hospital Society that he resorts when stricken down by accident or disease. A sailor's life is one of the hardest callings to which the destiny of man can wed him, and he is entitled, at the hands of those who stay at home, and of those who travel, to succour and assistance when in distress. The expenditure of the Society is over £20,000 annually, whereas the income has for several years fallen short of this amount. In connection with the Seamen's Hospital is the London School of Tropical Medicine, an institution which has already done the Empire incalculable service by advancing our knowledge of the causes and means of preventing diseases which are more or less peculiar to other climates than our own.

### HOSPITAL FOR SICK CHILDREN, GREAT ORMOND STREET.

THIS is probably the oldest children's hospital and to its successful development we owe most of the children's hospitals in the country. It has rendered great services to the poor of the metropolis. Last year it relieved 24,670 out-patients and 2,314 in-patients. During the last three years its average gross annual income has been £18,957, and its average gross annual expenditure £19,689, showing an annual deficiency of about £750. The number of patients benefited shows an alarming tendency to grow every year. Its especial needs are: (1) more income, and (2) a large sum to meet the expense of a modern electrical and scientific outfit.

It has the advantage of having on its Committee many men of high public reputation and ability, and its secretary, Mr. Adrian Hope, is one of the most active and successful of hospital secretaries.

### THE BIRMINGHAM GENERAL HOSPITAL.

THIS hospital was founded in 1766, and the new building, which was erected and opened in 1897, has one of the most attractive and striking elevations of any hospital in the country. It is administered by a committee which is without doubt one of the most active and capable bodies charged with the management of a great hospital. It should have an immense interest for musical people of high and low degree, for the Birmingham Festival had its origin in, and owes its success to, the circumstance that it is held every three years in aid of this great medical charity. The hospital contains 346 beds; of these 288 were daily occupied during last year, when 5,642 in-patients and 62,519 out-patients were relieved. In 1902 the total income from all sources was £19,768, and the total expenditure £25,405. This shows a deficit of £5,637. There was in addition a deficit of £5,111 brought forward from the previous year. The most pressing requirement of this hospital is a considerable increase in the income to maintain the institution in full working order, and to meet the large and increasing demands made upon it year by year. Few if any provincial hospitals are as well administered as the Birmingham General Hospital, and patients are received from all parts of the Midlands and from Wales.

### KING'S COLLEGE HOSPITAL.

THE present site of this hospital, which occupies an old burial-ground, has never been quite satisfactory. At the present time the necessity has arrived for rebuilding and extending. Among other requirements are a new nurses' home and a new out-patient department. As there is no room for expansion on the present site, and as there are other parts of London where the need of a general hospital is much greater, the authorities have made the very sensible decision to remove to a new district. The place chosen is Camberwell, where an anonymous donor has presented an area of 12 acres to the hospital. With this splendid site there can be little doubt that the new King's College Hospital when completed will be the most perfect and admirable institution of its kind in the country. Owing to the fact that Camberwell is a district which has hitherto been unprovided with a general hospital, and as it contains a very large number of the poorer classes among its residents, the sphere of usefulness of King's College Hospital is sure to be enormously increased by the move to fresh quarters. In order to defray the cost of the removal the authorities have issued an appeal to the public for £300,000. Seeing that there is such a brilliant prospect before the institution it is highly probable that all the money needed will be forthcoming. There is another aspect which makes it still more likely that this surmise will be fulfilled, and it is this, that King's College Hospital is particularly well administered in every respect. The secretary is Captain H. S. Tunnard.

### NATIONAL HOSPITAL FOR THE PARALYSED.

THIS hospital is an essential part of the hospital system in the metropolis, and in these days of hurry and strain every worker who has the means should make a point of giving something of his abundance in support of the National Hospital for the Paralysed. During 1903 the hospital has been honoured with the King's patronage and a Royal Charter of incorporation has been granted. It relieved 6,366 out-patients and 1,004 in-patients last year. The fact that with 200 beds only 1,004 in-patients should have been admitted for treatment emphasises the special character and difficulty of the work done by this Institution for the public of all classes. During the last three years the average gross annual income has been £14,183, and the average gross annual expenditure £17,177, showing a deficiency of about £3,000 per annum, for which an urgent appeal is made. Funds—especially new and increased annual subscriptions—are urgently needed for current expenses, as well as to carry out some necessary extensions both in the in- and out-patient departments. We hope that financial assistance may be forthcoming without delay, as it most certainly will be if the wealthier classes realise what the work of this hospital means for everybody and how pressing are its claims upon their generosity.

### UNIVERSITY COLLEGE HOSPITAL.

GOWER STREET, W.C.

THIS hospital stands in close proximity to a densely populated district, inhabited by working men and women, of whom many are of the very poorest class. Somers Town, than which there is probably no poorer or more squalid district in London, is in the immediate neighbourhood of the hospital. It is of the utmost importance to these people that the committee should be enabled to maintain the hospital and thus provide for the wants of the sick poor who flock to its doors. The number of beds at present available is 191, and the daily average number of in-patients during 1902 was 168, the total during the year being 2,693; also 46,856 out-patients and casualties were treated, their attendances amounting to the large total of 163,996. To deal with this enormous mass of suffering people, the ordinary expenditure, even with the most rigid economy, reached in 1902 the sum of £23,567, whilst the reliable income from all sources was only £8,000, thus leaving £15,567 to be contributed by the benevolent public. This institution has never attracted the financial support which its position as the hospital of University College ought to have secured for it long ago. It has the advantage of a new building (the generous gift of the late Sir J. Blundell Maple), now in course of erection on a somewhat novel plan, which is being equipped with the latest and most improved appliances. So soon as the whole of the old buildings have been demolished and the new hospital completed and opened it is estimated that fully £30,000 will be required annually to keep the enlarged hospital going. The pressing requirements this year are: (1) money to defray the cost of furnishing the new buildings; and (2) an additional income of £5,000 per annum at least. The committee earnestly urge upon all the necessity of contributing to the support of University College Hospital, offering on their part the assurance that no effort shall be wanting to ensure the economical administration of the funds placed at their disposal. Beds or cots may be endowed and named in perpetuity as desired. Payments for the purpose may be made in one sum or by instalments. Secretary, Mr. Newton H. Nixon.

### ROYAL FREE HOSPITAL.

The Royal Free Hospital has special claims upon the community as the first general hospital to which all classes of patients have from the outset been admitted free and without tickets. The example which it set at a time when the ticket system was rampant has yielded excellent fruit, for to day, with a very few exceptions, the chief passport to admission at the great London hospitals is, the urgency of the medical or surgical needs of each patient who presents himself for treatment. The Royal Free Hospital relieved 40,321 out-patients and 2,635 in-patients last year. During the last three years its average gross annual income from all sources, including legacies, has been £12,163, and its average gross annual expenditure £12,176. These figures afford the best proof of the economy and wise financial management of this hospital under the able guidance of Mr. Charles Burt, the chairman, who has worked most successfully and zealously on its behalf, and has been most ably seconded by Mr. Conrad W. Thies, the secretary. The pressing requirements of the hospital are: (1) structural alterations and the provision of new operating theatres, also of improved sanitary appliances and baths; (2) a new out-patient department; and (3) new floors throughout the hospital. An immediate gift of £10,000 and a considerable influx of annual subscribers are needed for this most deserving and excellent hospital.

### GREAT NORTHERN CENTRAL HOSPITAL,

Holloway Road, N.

The work of this hospital is annually increasing, and has now grown so large that the premises are in many respects found inadequate to the demands. This is especially so in the in-patient department, and for some time past considerable difficulty has been found in accommodating the increasing domestic and nursing staff. The committee are determined not to allow the hospital to fall behind in the race for efficiency, and the question of enlargement and extension is already under consideration. The great difficulty in the way is the want of funds, the utmost exertions being scarcely more than sufficient to make both ends meet. The hospital has to rely too much upon fluctuating sources of income such as legacies, and but for a certain amount of good fortune in regard to this item, the institution would be in serious difficulties; hence the prospect of increasing expenditure attendant upon enlargement is one which requires some courage to face. The administration of this hospital is sound, and has the advantage of one of the most able and successful of secretaries in Mr. Lewis H. Glenton Kerr. The number of in-patients last year was 1,941, and of out-patients 24,536.

### ROYAL NATIONAL HOSPITAL FOR CONSUMPTION.

This hospital consists of a number of separate houses or villas erected at Ventnor, Isle of Wight. It is conducted on what is known as the separate or home system, and has been widely popular in consequence. The number of in-patients under treatment last year was 907. The annual expenditure exceeds the assured income by £6,000. So long as consumption continues to prevail, so long should this institution continue to receive, as it deserves, liberal support from the public.

### CHARING CROSS HOSPITAL.

This institution has often been described as the central hospital for accidents in London. It has undergone considerable structural alteration, and a large sum is at present being expended upon new buildings, which are urgently necessary. It occupies the chief portion of a site isolated by streets off the Strand near Charing Cross, and for years public opinion has demanded that it should acquire the whole of this self-contained site, including that portion of it which is at present occupied by the Royal Westminster Ophthalmic Hospital. It relieved 18,073 out- and midwifery patients, and 1,986 in-patients last year. During the year the gross income has been £26,600 and the gross annual expenditure £49,000. This excess of expenditure over income represents the additional outlay on the new buildings. At the present time this hospital is appealing for £38,600 to make up the sum required for the current year and for the improvements. Its most pressing requirements are: (1) an additional £50,000 for the rebuilding fund, and (2) an increase of £5,000 a year to its income from voluntary sources. Mr. Arthur Reade, the secretary, who has long been connected with the institution, is one of the most popular personalities in the world of hospital officials.

### ST. LUKE'S HOSPITAL.

Founded in 1750 for the treatment of mental diseases, this hospital continues to carry on its important and charitable work. It ministers to the necessities of our large and deserving "middle-class" community of limited means, who are received either gratuitously, or upon a contribution towards the cost of maintenance, fixed according to the circumstances of each case. We understand that the Committee of St. Luke's are very desirous of removing to a better site in suburban London. The present site is held on perpetual lease from St. Bartholomew's Hospital on condition that it is maintained for the treatment of the mentally afflicted. We believe that negotiations are still being carried on between the two hospitals, and if they result in the removal of St. Luke's, the authorities of that hospital will be able to bring its work up to the best standard of medical science upon the treatment of insanity. Funds are much needed to keep this good work going, the necessary expenditure having exceeded the income for some few years past. The hospital is nearly always full to its capacity of 200 beds. Secretary, Mr. W. H. Baird.

### HOSPITALS WITH 100 BEDS AND UPWARDS.

### METROPOLITAN HOSPITAL.

THIS institution is situated in one of the poorest and most crowded districts of London. It deserves a much larger measure of support than it has heretofore succeeded in attracting. Of its 111 beds 92 were daily occupied during last year, and it relieved 1,285 in-patients and 40,592 out-patients. During the last three years the average gross annual income was £12,601, and the average gross annual expenditure £12,672. Some £20,000 are urgently required for a nurses' home in order to free for the reception of patients the wards now converted into cubicles. The secretary, Mr. Charles H. Byers, has for many years laboured assiduously and successfully to extend the resources of this important institution, which in the past has been far too little known to wealthy contributors to hospitals—a disadvantage which we hope will speedily disappear.

### LONDON FEVER HOSPITAL.

LIVERPOOL ROAD, N.

THE appearance of scarlet fever, diphtheria, or measles in a household causes the most intense inconvenience and anxiety to the householder. If, however, he has constituted himself a Governor of the London Fever Hospital, by payment of a small annual subscription, his difficulties will be relieved; as in every such emergency he is entitled to prompt help in the removal of the sufferer to this hospital, as well as to treatment free of charge for his domestic servants. Business houses, clubs, and hotels are accorded the same prompt help in case of need, with free treatment for certain of their employées. Many people subscribe to the hospital for the personal reasons indicated above, some from pure benevolence, and others in recognition of benefits received in the hospital either in their own persons or in the case of members of their households. All alike are entitled to the privileges sketched above; but, happily, comparatively few are obliged to use them in each year. The money thus saved enables the Committee to offer treatment to members of the general public who may be attacked by the diseases named, and who are able to pay about a fourth of their cost to the hospital. Over 1,500 persons have been received and treated in the last two years. They are people who decline to cast themselves upon the rates in infectious sickness, who are able to pay a part at least of the cost of their illness, and who if they remained for treatment in their own homes, could hardly fail to be a source of danger to all about them and to the community generally. There are private rooms for those who can pay approximately the full cost of their illness. This is the work of the London Fever Hospital, and during the last 100 years over 82,000 sufferers have been received. Who can estimate the number of lives that have been saved in all classes of society by a century of such work? Every household is interested in it. The diseases named are no respecters of persons; all are liable to their attacks. The most the householder can do is to prepare beforehand, so as to insure the prompt removal of the sufferer when the emergency may arise. Old friends are continually falling away, mostly by reason of death, and the Committee are compelled to seek new supporters. Assistance is therefore asked for this hospital which is in danger of languishing for want of additional help.

### MOUNT VERNON HOSPITAL.

PARTICULAR attention is drawn to the urgent need of completing the hospital at Mount Vernon, Hampstead, thereby increasing the accommodation from 100 to 140 beds. The duties of the medical staff have been of an exceptionally difficult and trying character. To be compelled to daily refuse applicants who are in every respect eligible for indoor treatment, and whose lives might thus be prolonged, is a sufficiently sad experience. But the difficulties do not end here. For those who are approved—and there are now nearly 250 such waiting their turn to be admitted—there remains the long and weary wait of at least twelve or fifteen weeks, during which time the disease which has attacked them pursues its course with such deadly effect that many who were not beyond hope of cure when approved, are hopelessly ill when they are received. It is to remedy these unsatisfactory conditions that help is required. Of the sum needed to complete the hospital, £10,000 has yet to be subscribed.—Secretary, Mr. W. J. Morton, who deservedly enjoys the confidence of his board.

### EAST LONDON HOSPITAL FOR CHILDREN.

FOR thirty years we have been inspecting hospitals of various kinds in all parts of the world, but we can truthfully say that every time we enter the East London Hospital for Children we are more and more impressed with the quality of the work it is doing for East London. To pass out from the gloom and squalor of the surrounding streets into the wards of this hospital is an altogether encouraging and surprising pleasure. Of its 135 beds 113 were daily occupied last year. It relieved 2,146 in-patients and 32,981 out-patients. During the last three years the average gross annual income has been £10,466, and the average gross annual expenditure £10,122, a proof of the economy and care with which the expenditure is controlled. Its most pressing requirements are (1) an isolation ward; (2) a nursing home; and (3) certain structural alterations. We hope that before long money for all three will be provided. Mr. Thomas Hayes has held the post of secretary for a good many years, and the institution has prospered and extended under his management.

### WEST LONDON HOSPITAL.

HAMMERSMITH ROAD, W.

OWING to representations which were made by King Edward's Hospital Fund for London, supported by the grant of £1,000 specially for the purpose, this hospital has had to undertake a heavy outlay upon the sanitary improvement of three of the old wards. Partly on this account and partly because of the wear and tear of the many years elapsed since the last extensive work of this kind was carried out, practically the entire premises, externally as well as internally, are being renovated, while, for sanitary and economic reasons, the installation of the electric light is being extended. These necessary improvements will entail an outlay of £5,000. Very slenderly endowed and dependent as the hospital is upon voluntary contributions, the board very earnestly appeal for help to meet this additional outlay. The works referred to have necessitated the temporary closing of about 50 of the 150 beds. Notwithstanding which 2,034 in-patients have been under treatment during the 10 months ended October 31, while, in the same period, 29,497 out-patients have also been relieved. As a centre for post-graduate teaching, this hospital has very considerably extended its popularity, for, while the number of fresh students enrolled throughout the year 1902 was 125, the number enrolled in the first ten months of this year is 180.

### CANCER HOSPITAL.

This hospital was founded in 1851 for the free treatment of the necessitous poor who are afflicted with cancer, tumours, or allied diseases. The nature of the disease makes it necessary to supply both food and dressings in great quantity and of high quality. The annual expenditure amounts to about £12,000, to meet which there are annual subscriptions of £2,000 and dividends £3,400. The balance has to be made up by donations, etc., and generally it means selling out stock to the amount of about £3,000 or £4,000. Recently the pathological department has been much enlarged, a photographic section being added, and an electrical department organised, and the treatment by Radium undertaken, all greatly adding to the efficiency of the hospital and also to the cost of maintenance. Secretary, Mr. F. W. Howell.

### ROYAL LONDON OPHTHALMIC HOSPITAL.

This hospital, like many other charities, has suffered severely in consequence of the war in South Africa. In 1899, when the new building was occupied, the committee confidently anticipated increased support from the public to meet the increased expenditure rendered necessary by the occupation of a larger building. The war began at that very time, and it has been difficult to collect new subscriptions and donations. In now pleading for help, the committee beg to point out that it is the earnest appeal of the managers of a great charity which has occupied an unique position in the kingdom for 100 years, and is now passing through a critical period which must be a turning point in the history of the oldest and largest eye hospital in London. Secretary, Mr. Robert J. Bland, City Road, E.C.

### LONDON HOMŒOPATHIC HOSPITAL.

At the festival dinner in aid of the hospital, which took place at the Hotel Cecil on June 25 last under the presidency of the Earl Cawdor, Vice-President and Treasurer, a munificent donor—"a nobleman" —not only promised £1,000 towards a total of £5,000, but added a promise of a further £1,000 if another £5,000 could be promised by Christmas. Captain Cundy also assented to double his subscription of £500. It is sincerely hoped that this munificent offer will not be allowed to go by forfeit, and that the many friends of the hospital will make a special effort to comply with the conditions, namely, that the promises be received by Christmas and the donations be received by June next year. The field of the appeals of this hospital is limited by the medical principle on which this hospital is established. The enormous area of general public benevolence is to a large extent out of its reach. The public, to whom the amount asked for would be a trifle, are without impulse to respond to the appeal. If it were possible to convey to a wider circle an impression of the manner in which the medical relief of the poor is carried on in the hospital, ample funds would readily be afforded. The Board can only look, therefore, to those who know the value of the medical principles which the London Homœopathic Hospital represents, and they venture to suggest it to be a personal duty incumbent on homœopathists to conserve their gifts in support of that hospital which others will not maintain. The immediate task in hand is to raise £12,000 to make good deficiencies of income during past years, which have necessitated the loan of £3,000 from the bankers and the withdrawal of £9,000 from the capital funds. Secretary-Superintendent, Mr. G. A. Cross.

### Hospitals with 50 Beds and Upwards.

### THE CHELSEA HOSPITAL FOR WOMEN,

Fulham Road, S.W.

This hospital, which has 50 beds, was founded in 1871 for the reception and treatment of respectable poor women and gentlewomen in reduced circumstances.

The council appeal for donations and new annual subscriptions (their only reliable source of income) in order to meet increasing demands without incurring a heavy debt, and to pay off a mortgage of £4,000 on the hospital. There is a convalescent home at St. Leonards-on-Sea, which contains 22 beds, and is open to others than those who have passed through the hospital. Secretary Mr. Herbert H. Jennings.

### THE HOSPITAL FOR WOMEN,

Soho Square, W.

There are 60 beds, all in constant use. The committee very earnestly appeal for additional annual subscriptions and donations for their maintenance. Secretary, Mr. David Cannon. This is one of the most comfortable hospitals in London from the patients' point of view.

### HOSPITAL OF ST. JOHN AND ST. ELIZABETH.

This institution, which has recently been rebuilt at 40 Grove End Road, is a hospital which ought to have the active and liberal support of all Roman

Catholics. It is most economically administered by Sisters of Mercy, and anyone who will visit it will be pleased and satisfied. Of 70 beds at present equipped 65 were daily occupied during last year, and 148 in-patients were relieved. These figures point to the fact that a large number of cases requiring prolonged treatment are taken in. During the last three years the average gross annual income has been £2,882, and the average gross annual expenditure £2,539. These figures must be regarded as approximate, because the hospital has been rebuilt within the period named, and had the beds all been available and occupied the expenditure would largely have exceeded the available income. From all we hear of this hospital we have confidence in commending it to the liberal support of the charitable.

### QUEEN CHARLOTTE'S LYING-IN HOSPITAL.

This hospital received 1,271 patients into its wards last year, and, in addition, attended 1,204 patients at their own homes. Although upwards of £25,000 have been spent during the past few years in enlarging and modernising the hospital and in building a nurses' home, so great have been the demands on the accommodation of the charity that the committee have resolved to proceed with an enlargement of the nurses' home at an early date. It is estimated that the cost will exceed £2,000, towards which contributions are earnestly solicited. Funds for general maintenance are also greatly needed, as, while the expenditure exceeds £5,000, the reliable income is but little more than £2,000. Donations may be sent to the secretary, Mr. Arthur Watts.

### BOURNEMOUTH NATIONAL SANATORIUM FOR CONSUMPTION AND DISEASES OF THE CHEST.

Patients in humble circumstances who are convalescent, who require further medical treatment and change of air, or who are suffering from an incipient form of the disease, are cases for the relief of which this institution was founded. The "open-air" treatment is carried out completely in all suitable cases. The National Sanatorium is at present in extreme need of help. It owes £500, and the necessary extension of the building and other improvements are delayed through want of funds, while the demands for admission are overwhelming, owing to the suitability of the climate of Bournemouth. All the 72 beds are occupied, and over 100 applicants are waiting for vacancies. The Committee of Management are making a special appeal for liberal support to enable them efficiently to continue and extend their work. Treasurer, G. J. Fenwick, Esq. Secretary, Mr. A. G. A. Major.

### POPLAR HOSPITAL FOR ACCIDENTS.

Blackwall, E.

Situated amongst a teeming population of poor hard-working people in a district which may be called the "workshop" as well as the "port" of London, this hospital is doing an excellent work. The demands on the institution have of late years greatly increased, and consequently further subscriptions and donations are very necessary. Secretary, Lieut.-Colonel Feneran.

### NORTH-WEST LONDON HOSPITAL.

This hospital, which was founded in 1878, is the only institution of the kind in the north-west district. It has 53 beds, and last year there were 601 in patients, and 43,674 attendances of out-patients. This institution has great claims upon all thoughtful persons from the circumstance that Mr. George Herring, who is probably the most beneficent of the regular contributors to metropolitan hospitals, takes an abiding interest in its upkeep and maintenance. There is great need for a large sum to enable the committee to get out plans and erect a modern hospital, so that the inhabitants of the north-west district of the metropolis may be adequately supplied with hospital accommodation. The prospect of extension is, however, rendered almost hopeless by the difficulty in providing funds for the present work, which entails a yearly expenditure of £4,500. Financially the hospital has had a bad year. At present the outstanding tradesmen's bills amount to over £2,600. We believe if this deserving hospital were more widely known it would not be thus crippled for want of funds. Secretary, Mr. Alfred Craske.

### Hospitals with Less than 50 Beds.

### HOSPITAL FOR EPILEPSY AND PARALYSIS.

This hospital has been rebuilt upon a new site in Maida Vale, and will shortly be opened for the reception of patients. We have far too few beds for the accommodation of patients suffering from paralysis and epilepsy and the numerous diseases of the nervous system, and a contribution towards the £31,000 required each year to maintain this institution would be well laid out. Mr. H. Howgrave Graham, the secretary, has devoted many years of his life to the work of financing this hospital under most difficult circumstances, and we hope that many charitable people will visit the hospital and identify themselves with its work.

### SAMARITAN FREE HOSPITAL FOR WOMEN,

Marylebone Road, N.W.

This hospital is entirely free. It is dependent upon the charitable public for support; it needs £6,000 per annum. Funds are wanted at once to meet outstanding liabilities. The committee appeal for bequests and donations towards a new endowment fund; £1,000 will endow and name a bed. £10,000 are required to build a new outdoor department, improve the accommodation for nurses, build a new operating theatre, etc. Secretary, Mr. W. G. King.

### WEST END HOSPITAL FOR DISEASES OF THE NERVOUS SYSTEM.

This hospital is doing a great and good work. The applications for treatment largely increase every year. The institution has no endowment or funded property whatever, and is entirely dependent upon the subscriptions and donations of the generous public to carry on its daily work. The need for a convalescent home by the sea, where the patients could be sent for a time after their discharge from the hospital, is a very pressing one, and the committee are striving hard to raise the necessary funds to carry out this scheme. Contributions towards this object will be gratefully acknowledged by the Secretary, Mr. Alfred J. Wise, at the Hospital, 73 Welbeck Street, W.

### BRITISH LYING-IN HOSPITAL,

Endell Street, W.C.

This hospital for married women has carried on its work in one of the poorest districts of London for 154 years. In 1898 it was found necessary to improve the accommodation for the staff and the patients, and an outlay of £2,000 was incurred. Considerable development in the relief afforded by this charity followed, and three years later two more wards were opened, and a nurses' home was built to accommodate the necessarily increased nursing staff. This caused an additional outlay of £7,000. The applicants for relief steadily increase, and the need for further extension to meet the enormously increased demands of the neighbourhood of St. Giles causes much anxiety to the committee. Less than one-third of the cost of the improvements has been subscribed by the public, and the invested funds have been sacrificed to meet the heavy expense. To replace some of these investments and to provide for the increased cost of maintaining this excellent hospital, subscriptions and donations are earnestly solicited. Secretary, Mr. A. C. Wickins.

### PADDINGTON GREEN CHILDREN'S HOSPITAL.

This hospital was established in 1883, and is supported entirely by voluntary contributions. It provides 46 cots. It is quite free, without letter of recommendation, to the children of the poor. 589 in-patients were admitted in 1902. There were also 15,011 new out-patients and 1,038 casualties and accidents, the total attendances of out-patients being 47,057. The hospital has a Convalescent Home, with accommodation for 16 children, at "Fair View," Slough, for patients who have passed through the wards. 117 children were admitted in 1902. Funds are greatly needed for maintenance purposes and also to pay off a debt of £2,400. Contributions will be thankfully received and acknowledged by the treasurer, George Hanbury, Esq., 28 Princes Gate, S.W., or Mr. W. H. Pearce, the secretary.

### MILLER HOSPITAL AND ROYAL KENT DISPENSARY.

Greenwich Road, S.E.

The Miller Hospital was the first general hospital erected in this country with circular wards. It is a comfortable little place, ministering to the needs of a large population in Greenwich and the neighbourhood. Of late years, however, the accommodation has been unequal to the demands of the densely populated neighbourhood in which the hospital is situated, and its enlargement is now an urgent necessity. The hospital has never attracted the support which it is entitled to receive from the inhabitants of Blackheath and the district which it serves, and now stands in pressing need of help to carry out the necessary extensions, and about £3,000 a year more income. The importance of the work it does will be seen from the statement that during last year 294 in-patients and 18,858 out-patients were relieved. Mr. James Marks, who has been the valued secretary for many years, will be glad to receive contributions in aid of the work to which he is devoted.

### HAMPSTEAD HOSPITAL.

A new hospital is in course of erection to meet the increased requirements of Hampstead, Highgate, and Kentish Town, but, to complete this necessary work further assistance to the extent of £12,000 is required. The old buildings contain 32 beds, and in the past eleven months 378 in-patients were relieved, while there were 6,577 attendances of casualty and out-patients. In spite of rigid economy in administration it is feared that there will be a deficit on the maintenance account for the present year of £500. To avert such an unfortunate contingency and to defray the cost of the new building is the immediate and anxious aim of its committee, and considering that a fair proportion of the residents of Hampstead and Highgate are in easy circumstances and might reasonably be expected to take a close interest in their own hospital, it is confidently hoped that the necessary help will soon be forthcoming. Secretary, Mr. George Watts.

### CITY OF LONDON LYING-IN HOSPITAL.

City Road, E.C.

The construction of a tube railway in close proximity to this hospital (which has been erected over 130 years) caused such serious damage to the foundations and structure that the committee, acting upon the advice of experts, sent in a claim for compensation against the railway company. After protracted negotiation and litigation the railway company admitted their liability, and made a payment of £3,000 in discharge of all claims. It then became necessary to ascertain the expense that would be incurred in restoring the old building and in carrying out necessary improvements, but it was found that the lowest tender amounted to so much more than the committee were advised to accept from the railway company that the question almost of necessity arose as to whether it would not be more prudent to entirely rebuild the old portions of the hospital rather than spend a considerable sum in partial restorations and attempted improvements of a building which, in the opinion of the medical staff, has been found for some time past unsuitable and inadequate to meet the requirements of modern medical science, the cubic space per patient being insufficient and the ventilation and hygienic arrangements obsolete, while a considerable part of the building stands on the ground, without any cellarage or arches. The subject was considered by a special Court of Governors on the 2nd inst., when it was wisely resolved to rebuild. This, however, will make severe inroads upon the capital of the hospital, which is made a much more serious matter by the fact that the income hitherto received from investments, amounting to £1,763, will be greatly reduced. By making these facts known it is felt that this old and necessary charity, which has been liberally supported for 130 years, will promptly receive such assistance as will enable it to carry out the works that have been forced upon it. Mr. R. A. Owthwaite, the Secretary, who has devoted most of his life to this and other charitable movements, will readily furnish full information regarding the improvements which have been decided upon and the expense that will be incurred thereby.

### CHARITY AND EFFICIENCY.

THE doctrine of efficiency possesses so severe a front that it is in many minds not readily accommodated to the quiet and modest virtue of charity. Its natural associations lie rather with the sterner claims of system, effort and discipline, guided in consort by judgment and marshalled with some degree of rigidity towards the attainment of a definite purpose in the pursuit of which secondary objects—themselves possibly laudable—must be ruthlessly pushed to one side. All this appears incompatible with, if not actually opposed to, the spontaneous impulse and ready emotion which are to many the inseparable accompaniments of the gentler virtues. The promptings of human sympathy and tenderness in response to a picture of misfortune and suffering are in most of us so natural and so entirely self-suggested that it almost seems ungracious to point out that they need regulation by the imposition of practical conditions. Still less welcome is the statement that apart from such conditions acts animated by the best of motives and performed in a spirit of true unselfishness may not only fail to attain a beneficent purpose, but may in fact and practice have a purely harmful result. Yet there are few, if any, social truths concerning which so unanimous a verdict is given by all those competent to speak on the matter than that the charity which consists in giving money or its equivalent repeatedly both misses its aim and produces results quite different from those intended by the donor—results actually prejudicial to the recipient and to the general cause of human wellbeing. It is not less but more necessary to emphasise this truth at a season when good-will is particularly active, and when the very abundance of its flow establishes a necessity for wise guidance.

The consideration that charity may thus run to waste, or worse, has an even more acute aspect when it is remembered that its total amount is a limited and more or less fixed quantity. At different times it may take different courses and flow in different directions, but when the period and area of observation are sufficiently extensive, it is found that the gross amount of money devoted to purposes of charity varies but little from year to year. Even some urgent and exceptional claim which receives widespread and substantial recognition hardly disturbs the normal average, for almost without fail one of its secondary effects is to curtail the amount flowing in other directions. From this it follows that an unwise charity not only fails in its purpose or even inflicts harm, but is in addition an actual loss of opportunity. It is so much subtracted from the limited sum available for the succour and assistance of suffering and unfortunate humanity, and it thus extends still further the melancholy catalogue of good that might have been. Failure in an intended purpose, the infliction of actual harm, and the prevention of successful service, constitute a severe indictment, and it is an indictment which has been established on repeated instances and on the most conclusive evidence against the practice of unregulated and undisciplined charity.

But if this is true there arises from it the conclusion that a mere sense of pity and the impulse of a benevolent disposition are not sufficient to translate the aspiration of charitable intention into successful action. There are also needed knowledge, judgment, common sense, and a wide outlook on the affairs of life. In a word, the qualities which secure efficiency in other departments of human activity must be enlisted in the service of charity if she too is to deserve the respect due to those who fight a good fight and contribute something to the success of human endeavour.

These it must be acknowledged are apt to be regarded as hard sayings when presented to the individual benefactor. He is so certain of the sincerity of his motives, the immediate position seems so obvious an opportunity of doing good, and the direct effect of his action appears so clearly beneficial, that he is naturally apt to discard warnings of possible or probable harm. Indeed he not infrequently resents such warnings as the unwarranted interference of an impertinent officialism and meets them with the protest that he has a right to do what he will with his own. The question of legal right is obviously not in dispute, but manifestly no man has a moral right to inflict ill upon others or to offer himself as an example of unwise or unworthy conduct. Indeed the more conspicuously he has the opportunity of illustrating his theories by action the more urgent it is not only that his motives shall be benevolent but that his conduct shall be wise. Generosity is so chivalrous an attribute that it may well escape a severely critical analysis. All the more therefore when presented in a pronounced fashion is there a moral demand that it shall be exercised in a manner calculated to produce benefits and not injuries. Those who command a long purse equally with those of narrower means are thus under the same dispensation. It is with the one class equally with the other to exercise charity with wisdom and judgment. On the one there is the special claim provided by the widespread influence of a prominent example, on the other the consideration that the more scanty the opportunity the greater the need that it be employed to full purpose.

In thus urging the necessity for the direction of charity by knowledge and reflection it is by no means suggested that the virtue should be practised only through the channels of special organisations. For individuals who cannot or will not spare time to investigate facts this may be the safest and wisest course. Without question it is to be preferred to the loose, unsound, and essentially illiberal practice of marking off so much for charity as a sop to conscience, and giving this in divided amounts to the first persons who submit an appeal. But no distributing organisation or system of official inquiry can equal the efficacy of the really earnest individual who is able to collect for himself the facts necessary to guide his bounty. It is the charity so practised which is indeed "twice blessed."

The need for efficiency, as above urged, would appear to make the practice of successful charity a more arduous undertaking than is generally allowed. This may be admitted. It is no more easy to achieve a wise ideal in charitable than in other purposes. But there are aspects of the discussion that present more comforting reflections. For example, if it is true that charity to be successful must be organised and directed by those qualities which promote efficiency, it is manifest that anyone who can contribute these qualities may render not less valuable service than the donors of money. Now it is notorious that public moneys do not always receive that scrupulous supervision and care which lie at the basis of true economy, and this is apt to be equally true of the management of charitable institutions. Hence there is abundant room for valuable personal service in this direction. Officials and organisations often resent criticism, but enormous benefits have frequently been secured for the recipients of charity by a vigorous scrutiny of those charged with its distribution. Business methods do not as a rule find a stimulating atmosphere in a board of gratuitous governors, and there is danger of the statutory meetings becoming mere opportunities for the registration of the decisions of the paid officials. There is thus in connection with the management of hospitals and similar institutions an opportunity for serving the cause of charity in no halting or feeble fashion. No consideration so surely stays the hand of the wisely benevolent as the suspicion that his money may be improperly administered. Again and again one hears of the refusal of contributions to particular institutions on the ground that the management is inefficient. It is not suggested that there is anything in the shape of fraud or bad faith, but only that the financial organisation is not conducted on business lines. That, if correct, is sufficient, and rightly sufficient to check the intending contributor. On the other hand, evidences of sound financial methods and good business management are most powerful influences in determining the flow of subscriptions, and he who can secure and enforce these is indeed a true disciple and servant of the sacred cause of charity. Such service may not command the attention paid to a large cheque, and to the sentimental may appear dull and prosaic. But it is real and enduring, and those who proffer it have this substantial triumph, that they adorn the golden crown of charity with the jewels of thoroughness and efficiency. This merit of personal service is in some form or other open to all, and those who neglect or refuse it deny the claim of human brotherhood and banish from their own lives opportunities for moral self culture which "lie scattered at the feet of man like flowers."

### THE GENERAL CHARITIES.

### ROYAL BLIND PENSION SOCIETY.

This society provides pensions by monthly instalments for blind people. The number thus assisted is 1,106. Further contributions are greatly needed, particularly annual contributions, to enable the committee to extend the society's pensions to the many applicants whose circumstances have been investigated and their worthiness testified. The income requires to be increased by at least £1,500 annually. Secretary, Mr. W. E. Terry, 237 Southwark Bridge Road, S.E.

### IRISH DISTRESSED LADIES FUND.

Relief is given by this society independently of any question of politics or religion. It also, if possible, finds employment for those who are able to work, affords pecuniary assistance to the aged and infirm, and pays for the education of children. There are 69 pensioners (mostly resident in Ireland) on its books this year, one having reached her 100th year, and 108 ladies are helped to earn their living through the work depôts in London and Dublin. The Secretary is General W. M. Lees, 411 Oxford Street, W

### ASSOCIATION FOR THE ORAL INSTRUCTION OF THE DEAF AND DUMB.

This association is entitled to the credit of being the first to publicly introduce into the United Kingdom the German, or pure oral system for teaching deaf and so-called dumb children to speak *viva voce* and to understand the spoken words of others by lip reading. The expenses, which are very heavy, are met by voluntary contributions and fees, which fall short of the amount imperatively needed to the extent of about £600 per annum. Lord Avebury is the Treasurer, and the Director is Mr. William Van Praagh, 11 Fitzroy Square, W.

### ORPHAN WORKING SCHOOL.

Haverstock Hill, N.W., and Hornsey Rise, N. Offices, 73 Cheapside, E.C.

A national and undenominational institution which maintains 500 children, varying in age from infancy to fourteen or (in special cases) fifteen years. It is greatly in want of funds at the present time. Appeal is made specially for new annual subscriptions, the normal income of the charity being more than £2,000 below what is needed to meet the expenditure. Secretary, Mr. Alexander Grant.

### THE NATIONAL REFUGES AND TRAINING SHIPS.

Headquarters—164 Shaftesbury Avenue, London, W.C.

This society, which has now been established 60 years, has eight homes on shore and two training ships on the Thames. Nearly 17,000 poor boys and girls have been trained for useful service. The work is supported by voluntary contributions, and no votes or elections are needed to secure the admission of any child. A special effort is now being made to raise funds for providing a new home for little girls in commemoration of the society's sixtieth year. The secretary, Mr. H. Bristow Wallen, will gladly give full information on this subject to any of our readers needing the same. It is satisfactory to know that this old established charity is vigorously maintained and that such beneficent work is absolutely free of debt.

### THE CHILDREN'S HOME.

Bonner Road, N.E.

This institution, or group of institutions, may fairly claim to take rank among the larger national charities, inasmuch as the children are received from various denominations, irrespective of creed, the destitute condition or moral peril being the first essential to a child's admission. Founded by Dr. T. Bowman Stephenson in 1869, with branches in various parts of the country, the Home has sheltered 6,000 children. When Dr. Stephenson retired in 1900, his place as principal was taken by Dr. Arthur E. Gregory, and rapid strides have since been made. The heavy burden of debt which existed a few years ago has been removed, and the income has reached a higher total than ever previously. There are at present 1,500 children in the Home, an increase of 170 upon the numbers at the corresponding time last year. The parent establishment is at Bonner Road, N.E., the Farm Colony is at Edgworth, in Lancashire, the Princess Alice Orphanage, near Birmingham, and the certified Industrial School for Boys is at Farnborough (Hants). Then there is the Convalescent Home and Cripples' House at Alvestoke (Hants), a small branch at Ramsey (Isle of Man), and Cottage Homes at Chadlington (Oxon ). A further development is the opening of a home at Chipping Norton (Oxon.) for crippled and afflicted children. A new branch, the gift of Miss Fowler, of Liverpool, will shortly be opened at Frodsham. Last, but not the least important is the Emigration Home for boys, in Canada. A special feature is the home life of the children, who are gathered into small communities, each with its own family circle, and its own interests, under the care of a "Sister-of-the Children." A good elementary education, useful industrial training (for boys) and domestic service (for girls) are given to all the children. To support such a rapidly-growing work a steady growth in the income is necessary. Unfortunately, the income for the present year is seriously deficient, and the need for funds is urgent and extreme. £16 will support a healthy child for one year, and £30 will provide for a cot in one of the hospitals or the Cripples' Home for a like period. All communications and remittances should be sent to Rev. Arthur E. Gregory, D.D.

### CLERGY ORPHAN CORPORATION.

This society provides education, etc, in its schools at Canterbury and Bushey for the orphan sons and daughters of clergymen of the Church of England. Many of the boys and girls proceed to the universities and colleges for women with exhibitions and scholarships founded by benefactors of the Corporation. An "Apprenticing Fund" enables the committee to assist the boys and girls when they leave school to make a start in life. The voting system in connection with the admission of children into these schools has now been abandoned. Every qualified applicant during the last five years has been admitted ; none have been excluded. (1) Owing to the adoption of this policy the numbers in the schools have risen from 228 to 256 ; (2) anxiety, disappointment and expense, inseparable from the voting system, have been spared to the mother and friends of the candidates ; (3) every boy and girl now admitted can expect two years more of school life than was possible for those who had to wait for success at contested elections. To make this policy a financial success an income from voluntary sources of £8,500 (annual subscriptions, donations, and church collections) is urgently needed. The secretary is the Rev. Wm. Chas. Cluff, M.A., 35 Parliament Street, Westminster, S.W.

### LONDON LOCK HOSPITAL AND RESCUE HOME.

Help is required for this valuable institution. There has been a continual flow of patients to the Hospital, some of whom have found their way to the Home and other homes, in many cases with very good results. It is the desire of all connected with the Hospital and Home to do the utmost in their power to succour those who are in want of a helping hand. Secretary, Mr. A. W. Cruickshank.

### FREE CONVALESCENT HOMES.

The Metropolitan Convalescent Institution meets the necessities of those among the sick poor who, after treatment in the hospitals of the Metropolis or elsewhere, require their restoration to health and strength by the aid of fresh air and a wholesome nourishing diet, which they are unable to obtain in their own scantily provided, and often unhealthy, dwellings. To take up cases where the hospitals and dispensaries leave them, to complete cures which physicians and surgeons have so well begun, is the commendable function of the charity. The charity, which was the first of the kind established in this country, commenced its beneficent work 63 years ago. Its operations have been steadily extended until now it maintains four homes, containing 541 beds, to which over 6,400 patients have already been admitted this year, for a period of three weeks, entirely free of charge. Since 1840 no fewer than 153,992 adults and 35,762 children have been received, the greater number of whom were fully restored to health and strength. In spite of strict economy, the cost of maintaining the four homes is about £13,000 a year, nearly all of which has to be obtained from voluntary sources, and a very earnest appeal is made for additional annual subscriptions and donations, so that the great and important work of the charity may be continued unimpaired. The Board feel that every succeeding month and year since the first opening of the institution has, in the progressively increasing number of applicants, and in the proportional amount of good conferred upon them, not merely afforded a triumphant vindication of the soundness of the principles upon which it was founded, but has firmly established its claim to be regarded as one of the very best and most important charities. The Secretary, Mr. Alex. Hayes, whose zeal and ability have contributed largely to the progress of this charity will gratefully receive subscriptions and donations at the office, 32 Sackville Street, W. The Treasurers of the Institution are Lord Haliburton, G.C.B., and James S. Strange, Esq.

### THE MARY WARDELL CONVALESCENT HOME FOR SCARLET FEVER.

This is the only Home for Convalescents from scarlet fever, which is always more or less rife in the Metropolis. There is urgent need for £2,000 to repay a loan which had to be raised for the purpose of defraying the cost of relaying the entire drainage system to meet the requirements of the District Council and to clear off other debts. The repayment of the loan is being pressed for, and the Home is absolutely without funds. Donations and subscriptions may be sent to Miss Mary Wardell at the Home, Stanmore, Middlesex, or to Messrs. Barclay and Co., Limited, 1 Pall Mall East, S.W.

### LONDON ORPHAN ASYLUM, WATFORD.

The managers plead for subscriptions and donations towards the £13,600 required from voluntary sources. Orphans from all parts of the British Empire are eligible, and since the formation of the institution in the year 1813 no fewer than 6,500 boys and girls have been sheltered within its walls. That the children are well cared for and trained no one who has visited the institution can doubt. Forty other children, viz., 25 boys and 15 girls, are to be received in January. A glance at the list of candidates reveals the national character of the charity, as applicants are there not only from all parts of England, but also from Capetown, Buenos Ayres, Australia, and Bombay. These are not only children of master tradesmen and clerks, but among others we see the father's calling was that of a master mariner, veterinary surgeon, farmer, journalist, engineer, dentist, chemist, assistant overseer, etc. Work performed on this broad-minded and generous plan should appeal to all. Yet it lacks the direct claim which every trade or class institution has upon its own distinct section of the community. The managers justly plead that its cosmopolitan character should elicit support on a very comprehensive scale, and subscriptions and donations will be gladly received by the secretary, Mr. Henry C. Armiger, 21 Great St. Helens, E.C.

### THE CHRISTIAN COMMUNITY.

For 200 years the members of this venerable society have been engaged in taking the Gospel to the very lowest classes found in common lodging-houses and the slums of the Metropolis, also to the aged and infirm in the workhouses. While mindful of the spiritual needs of the masses care has also been taken to look after their bodies as well. In this way various benevolent agencies have been carried on by which a vast amount of misery has been alleviated. The labour has been given gratuitously, so that whatever financial assistance is sent is used for the object it is intended to benefit. The committee are anxious to secure annual subscriptions, as there is no endowment, and they greatly feel the need of a more reliable income. Secretary, Mr. Jas. Atkinson, The Hall, London Street, Bethnal Green, E.

### FIELD LANE RAGGED SCHOOLS AND REFUGEES FOR THE HOMELESS POOR.

This institution was founded, under the auspices of the late Lord Shaftesbury, in 1841. Since that time the work of the charity has grown to great dimensions, and the scope of its activities has considerably expanded. It now includes a crêche for babies, an industrial home for boys, an institute for young men, a club for young women, band of hope and temperance society, refuges for men and women of character, and all the organisations of a large and important mission, by means of which many thousands are benefited weekly. To maintain the work in a state of efficiency £7,500 per annum are required; towards this sum only £2,000 to £3,000 is secured to the industrial home, leaving £4,500 at least to be raised by voluntary contributions. The committee have been compelled to obtain an overdraft from the bankers amounting to £3,000, owing to heavy expenses in connection with sanitation and special repairs at the boys' school and refuges, and the great difficulty in obtaining contributions, owing to the present financial depression. An earnest appeal is made for financial assistance to defray this expenditure. Voluntary workers are needed to aid in carrying on and developing this work amongst the poor. Subscriptions and donations should be addressed to the Treasurer, Field Lane Institution, Vine Street, Clerkenwell Road, E.C.

